# Association between Blood Group and Change in Coagulation Factors in Plasma Preparations for Transfusion Purpose at Kisii Teaching and Referral Hospital

**DOI:** 10.1155/2023/3749773

**Published:** 2023-11-09

**Authors:** Collince Odiwuor Ogolla, Benson Nyanchongi, Rodgers Norman Demba

**Affiliations:** ^1^Department of Applied Health Science, School of Health Science, Kisii University, P.O. Box 408-40200, Kisii, Kenya; ^2^Department of Medical Laboratory Science, School of Medicine, Maseno University, P.O. Box 3275-40100, Maseno, Kenya

## Abstract

**Background:**

Blood component therapy helps in managing patients with reduced hematopoiesis, elevated peripheral destruction of cells, and generalized blood loss (bleeding). Increased prevalence of arterial and venous thrombotic disease linked to the impact of ABO blood group on plasma levels of coagulation glycoprotein is demonstrated by blood group non-O persons.

**Objective:**

This study had a main objective of determining the association between blood group and change in coagulation factors in plasma preparation for transfusion purpose.

**Methods:**

The study employed a longitudinal study design. Factor assay evaluation was done by the use of Erba Mannheim ECL 105 semiautomated coagulation analyzer from India. Thawing meant for consequent coagulation factor analysis and sequential testing of stored cryoprecipitate and fresh frozen plasma was performed by the use of Stericox plasma thawing bath before being analyzed by the coagulation analyzer. Blood group of the collected blood sample in purple EDTA vacutainer was analyzed using blood antisera and a clean white tile, and results were recorded which helped in establishing the association existing between plasma and blood group. The data were fed into Excel and were evaluated by the use of SPSS version 25.

**Results:**

There was no significant association between coagulation factors in fresh frozen plasma and blood group, coagulation factors in cryoprecipitate plasma and blood group of the donors showed that the relationship was not significant with, (*r* = −0.116, −0.097, 0.007 and 0.047 with *p* value (0.900, 0.087, 0.096 and 0.096), respectively, which are greater than 0.005 standard alpha value.

**Conclusion:**

This study has shown no significant association existing between blood group and change in coagulation factors in plasma preparations at Kisii Teaching and Referral Hospital.

## 1. Introduction

High levels of plasma of several coagulation factors are linked with higher risk of venous thrombosis [1]. However, the mechanisms essential for these associations and the ones which take part in the control of plasma levels of coagulation factors are unknown [1].

Blood group non-O persons exhibit increased incidence of venous thrombotic disease and arterial disease compared to group O persons [2]. This risk is related to the point that ABO blood group impacts the levels of von Willebrand factor. The levels of von Willebrand factor are 25% greater in non-O groups when related to group O persons [2].

ABO blood group defines plasma von Willebrand factor levels in a mechanism which has not been determined [3]. An association existing between plasma von Willebrand factor and ABO blood group and coagulation factor VIII levels has not been well known. Blood group non-O individuals show significantly increased threat of arterial and venous thrombosis [4], whereas blood group O persons manifest hereditary bleeding tendency and vW disease [5].

In a previous study conducted on fresh frozen plasma (FFP) and blood group [6], a total of seventy-five plasma samples were obtained from 25 blood donors which were further classified into 3 groups: Group A (fresh frozen plasma), Group B (leukocyte filtrated fresh frozen plasma), and Group C (plasma frozen within 24 h—FP24), prothrombin time, international normalized ratio (INR), activated partial thromboplastin time (aPTT), factors IX, V, VIII, VII levels and fibrinogen were coducted for all samples and also comparison of coagulation factors levels in fresh frozen plasma in various blood groups. The results showed significant dissimilarity between INR, PT, and aPTT with *p* ≤ 0.001 less than 0.05 standard alpha value [6]. Factor VII had significant dissimilarity with*p*value (0.03) existing between the three groups, fresh frozen plasma showed a significantly greater level of factor VII when related to filtrated fresh frozen plasma (98.92 vs. 82.52%) with*p*value (0.02). There was no significant dissimilarity between FFP and PF24 detected with*p*value (0.76) greater than 0.05 standard alpha value [6]. Factor VIII had significant dissimilarity with*p* ≤ 0.001 between the three groups, Filtrated FFP and FFP which had no significant dissimilarity in regard to the level of FVIII with*p*value (0.72) which is greater than 0.05 standard alpha value. FFP significantly had a greater level of FVIII when related to PF24 with *p* value less than 0.05 standard alpha value [6]. FVIII was statistically greater in non-O blood groups with *p* value of 0.03, whereas other factors had no statistical differences with *p* value less than 0.05 standard alpha value. The leukocyte filtration of FFP was found to have no effect on the majority of coagulation factor activities although FVII level was found to be decreased, which is still sufficient for surgical hemostasis. The PF-24 which caused reduced levels of FVIII and fibrinogen with no significant changes in FIX, FV, or FVII is recommended for FFP indications that specifically require replacement of FVIII or fibrinogen in cases of Hemophilia. The study found no significant dissimilarity in coagulation factors of fresh frozen plasma between blood group O and blood group non-O [6].

Coagulation factors are confirmed by determining the factor's activity level in the plasma. Activity assays normally detect reduced levels of protein or proteins that are not functioning properly. Coagulation factor antigen investigations tell how plentiful the protein is available, but not whether its functioning is normal [7]. Clotting assays are grounded on the capability of plasma tested to correct the lengthy clotting time of plasmas with known factor deficiencies [7].

Levels of coagulation factors are mainly measured in clinical laboratories by assays which are based on the capability of test samples to correct APTT (for FXI, XII, VIII, and IX) or the PT (for FVII, FX, FV, and FII) [8].

Functional defects which are not detected by antigenic and amidolytic assays are detected by coagulation assays. The consequences resulting from failing to detect a sporadic functional defect by amidolytic assay must be balanced against generally poorer precision and the poorer specificity of coagulation assays [8]^.^

## 2. Methods

### 2.1. Study Site

Kisii Teaching and Referral Hospital Hematology Laboratory Department was the study site for this current study. The hospital is located within Kisii town, Kisii County, Kenya.

### 2.2. Sample Size

The study included one hundred and eight volunteer donors.

### 2.3. Study Design

Cross-sectional study design with time series analysis of cryoprecipitate and fresh frozen plasma stored at minus 18°C for up to five weeks with an interval of one week was employed for this study. Quadruple blood bags containing citrate-phosphate-adenine anticoagulant were used to collect 450 ml blood for subsequent processing into fresh frozen plasma and cryoprecipitate for storage at minus 18°C. The collected blood within 8 h was balanced and subjected to a centrifugation of 4000 rpm for nine minutes which generated about 180 ml plasma. The plasma was then separated and aliquoted into 3 parts each containing sixty millimetres. The first aliquot was used to evaluate changes in coagulation factors in FFP and in cryoprecipitate plasma at room temperature at baseline during week one (baseline). The second aliquot was used to evaluate the changes in coagulation factors in FFP and in cryoprecipitate plasma stored at −18°C after 3 wks of storage. The last aliquot was used to evaluate the changes in coagulation factors in FFP and in cryoprecipitate plasma stored at −18°C after 5 wks of storage. Analysis of coagulation factors was done by the use of Erba Mannheim ECL 105 coagulation analyzer. Stericox plasma thawing bath was used to thaw FFP and cryoprecipitate for subsequent serial testing and coagulation factor analysis at 37°C for forty five minutes, and results were recorded. Homogenicity was ensured by monitoring and maintaining proper standard storage conditions for the aliquots.

Blood sample collected in purple EDTA vacutainer was analyzed using blood antisera and a clean white tile to determine the blood group of the donor, and results were recorded which helped in establishing the association between blood group and plasma.

### 2.4. Data Management and Statistical Analysis

Statistical Package for the Social Sciences (SPSS) software version 25.0 was used to analyze data. The value measured data was recorded as numbers. The raw data collected from the study were entered in Microsoft Office Excel spreadsheet before being transferred to SPSS. The statistical analysis employed included descriptive statistics. Tables and graphs were used to present the findings from the analysis.

### 2.5. Ethical Considerations

Ethical clearance for this study was acquired from Baraton Ethical Review Committee. Research permit for the study was acquired from National Commission for Science and Technology (NACOSTI).

## 3. Results

### 3.1. Demographic Data

This study included one hundred and eight eligible participants, where all were volunteer blood donors who helped in achieving the objective of the study.  Table1 presents the dissemination of the blood donors evaluated as per their blood group.

Majority of the respondents, 33 (30.6%), were blood group A positive, followed by those who had blood group O positive with 29 (26.9%). The other remaining four blood groups that's; B positive had 22.2%, AB positive 15.7%, O negative 2.8% and A negative with 1.9% distribution in decreasing order respectively as illustrated in Table 1above.

**Figure 1:**
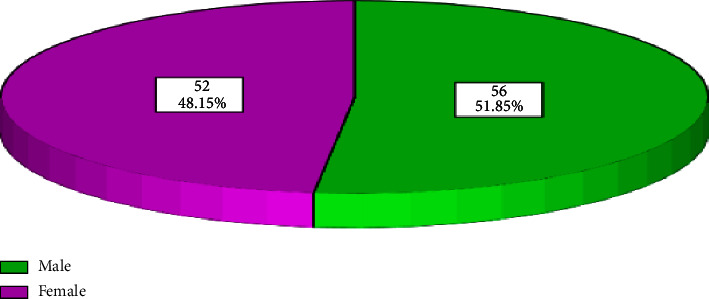
Pie chart showing distribution of respondents as per gender.

Majority of the participants were male at 56 (51.85%) while female donors were 52 (48.15%). This implies that the study was dominated by male respondents as compared to females as shown in Figure 1.

From Table 2, the findings from the analysis indicate the mean of coagulation factors in cryoprecipitate on week one represented with CROPW1 as 119.10. Week three had a mean of 109.81 while week five had a mean of 100.28. The standard deviation for the weeks was 19.6, 19.22, and 18.83, respectively. This indicates that there was no wide variation in coagulation factors for the five weeks but were distributed within the central value which is the mean of one hundred and eight participant. From the mean values and standard deviation values it was clear from this analysis and findings that there existed a significant difference in coagulation factors for the storage period hence confirming the normality aspect of the data.

### 3.2. Friedman's ANOVA Test Analysis for Coagulation Factors in Cryoprecipitate Plasma

Mean rank test of Friedman's test analysis was used to determine the differences in time period for coagulation factors in cryoprecipitate plasma, and the findings are shown in Table 3.

The mean rank for cryoprecipitate as shown in Table 3 on the first week (CRYOPW1) to fifth week (CRYOPW5) was in a reducing trend with 3.000, 1.990, and 1.010, respectively. This reveals that there is a firm significant difference existing in mean ranks for the period in a reducing trend, and hence the coagulations factors diminish with time.

### 3.3. Friedman's ANOVA Test Analysis on Changes in Coagulation Factors in Fresh Frozen Plasma

Friedman's test analysis was used to determine the changes of coagulation factors in FFP by the use of mean rank test. The findings are presented in Table 4.

As illustrated in Table 4, the mean rank for FFP on the first week represented by FFPW1 was 3.000, 2.000 for the 3rd week after storage, and 1.00 for the 5th week [9]. This confirms a significant difference existing in mean ranks in a declining trend.

### 3.4. Analysis of the Association between Blood Group and Change in Coagulation Factors

To determine the association, the study employed Spearman's rank correlation statistic method. The parameters used to analyze both the coagulation factors in FFP and cryoprecipitate were based on the time of storage of blood from week one to week five at −18°C.

The findings for week one presented in Table 5above about Spearman's Rank Correction for Association between Blood Group and Coagulation Factors in FFP shows that there was a week positive association but not significant with, (*r* = 0.039, *p*value (0.114).

Results of the third week indicated that the blood group had a week positive correlation influence on the coagulation factors in FFP, but the influence was not significant with (*r* = 0.029, *p*value (0.110).

The fifth week of the study showed a weak positive correlation which was not scientifically significant as shown by (*r* = 0.115, *p*value (0.095). The results indicated the same outcome from the results of the first two tests.

The results of week one presented in Table 6above about Spearman's Rank Correction for Association between Blood Group and Coagulation Factors in Cryoprecipitate Plasma shows that there was a weak negative association which was not significant with, (*r* = −0.116, −0.097, 0.007 and 0.047 with*p*value (0.090, 0.087, 0.096 and 0.047, respectively) which is greater than 0.05 standard alpha value. Thus, the results were not statistically significant since the*p*value (0.090).

Results in week three showed that blood group of the blood donor has a weak positive influence on the coagulation factors in cryo with (*r* = 0.007, −0.093, −0.093 and −0.050 with*p*value (0.096, 0.062, 0.062 and 0.092), respectively, which is greater than 0.05 standard alpha value. Hence, the association was scientifically not significant considering the fact that the probability value of 0.096 for the first factor for the third week was greater than 0.05.

Results of the last week of the study found that there was no relationship between blood group and coagulation factors in cryoprecipitate plasma with (*r* = −0.163, −0.050, −0.132 and 0.005) and*p*value (0.060, 0.092, 0.061 and 0.096) respectively.

## 4. Discussion

This current study observed that there was no significant association between coagulation factors in FFP and blood group of the donors. However, Wang et al. [10] reported that there was a lower significant association between blood group and the coagulation factors in FFP. The most likely explanation to the discrepancy could be attributed to the fact that Wang et al. [10] stored their sample at −70°C and the metabolic rate of the cell usually decreases when stored at negative temperature for an extended period.

The results for the third and fifth week were also not statistically significant for coagulation factors in FFP with (*r* = 0.029, *p*value 0.110; *r* = 0.115, *p*value (0.095). A study by Chai-Adisaksopha et al. [11] also affirmed no significant association existing between the blood group of the blood donated and coagulation factors in FFP.

The likely explanation as to why coagulation factors in FFP and blood group of the donors were not statistically significant could be attributed to the temperature at which the blood is stored before transfusion after blood donation. The results from this study agree with Philip et al. [12] who reported that the blood groups and coagulation factors in cryoprecipitate plasma were not significantly associated.

The findings of this study also mirrored the findings of Azad et al. [13] who observed no significant difference on blood groups AB, A, and B concerning coagulation FVIII.

## 5. Conclusion

The study has shown no significant association existing between blood group and change in coagulation factors in plasma prepared for transfusion purpose at KTRH. These new data are very essential in the management and diagnosis of venous thrombosis disease.

## Figures and Tables

**Table 1:** Distribution of respondents as per their blood group.

Blood group	Frequency	Percentage (%)
O positive	29	26.9
A positive	33	30.6
B positive	24	22.2
AB positive	17	15.7
O negative	3	2.8
A negative	2	1.9
Total	108	100.0

**Table 2:** Descriptive statistics for coagulation factors in cryoprecipitate plasma.

Variables	*N*	Mean	Std. deviation	Minimum	Maximum
CRYOPW1	108	119.10	19.96	61.75	157.25
CRYOPW3	108	109.81	19.22	56.00	148.25
CRYOPW5	108	100.28	18.83	52.50	138.50

**Table 3:** Friedman's test for mean rank.

Variables	Mean rank
CRYOPW1	3.000
CRYOPW3	1.990
CRYOPW5	1.010

**Table 4:** Friedman's test for mean rank on changes in coagulation factors in fresh frozen plasma.

Time in weeks	Mean rank
FFPW1	3.000
FFPW3	2.000
FFPW5	1.000

**Table 5:** Spearman's rank correction for association between blood group and coagulation factors in FFP (*N* = 108).

		Blood group						Spearman's correlation	
		O positive	A positive	B positive	AB positive	O negative	A negative	Correlation value	*p* value
Week 1 FFP	Normal	25	29	24	14	3	0	0.039	0.114
	Abnormal	4	4	0	3	0	2		

Week 3 FFP	Normal	24	27	24	13	3	0	0.029	0.110
	Abnormal	5	6	0	4	0	2		

Week 5 FFP	Normal	16	15	12	6	1	1	0.115	0.095
	Abnormal	13	18	12	11	2	1		

**Table 6:** Spearman's rank correction for association between blood group and coagulation factors in cryoprecipitate plasma (*N* = 108).

		Blood group						Spearman's correlation	
		O positive	A positive	B positive	AB positive	O negative	A negative	Correlation	*p* value
W1F1	Normal	27	29	23	16	3	0	−0.116	0.090
	Abnormal	2	4	1	1	0	2		

W1FVIII	Normal	29	31	24	17	3	0	−0.091	0.087
	Abnormal	0	2	0	0	0	2		

W1FXIII	Normal	29	32	22	17	3	1	0.007	0.096
	Abnormal	0	1	2	0	0	1		

W1 von Willebrand	Normal	28	30	23	17	3	0	0.047	0.096
	Abnormal	1	3	1	0	0	2		

W3F1	Normal	29	31	24	16	3	1	0.007	0.096
	Abnormal	0	2	0	1	0	1		

W3FVIII	Normal	29	33	24	16	3	2	−0.093	0.062
	Abnormal	0	0	0	1	0	0		

W3FXIII	Normal	29	33	24	16	3	2	−0.093	0.062
	Abnormal	0	0	0	1	0	0		

W3 von Willebrand	Normal	29	32	24	17	3	0	−0.050	0.092
	Abnormal	0	1	0	0	0	2		

W5F1	Normal	29	32	24	16	3	1	−0.163	0.060
	Abnormal	0	1	0	1	0	1		

W5FVIII	Normal	29	32	23	16	3	2	−0.050	0.092
	Abnormal	0	1	1	1	0	0		

W5FXIII	Normal	28	33	24	16	3	2	−0.132	0.061
	Abnormal	1	0	0	1	0	0		

W5 von Willebrand	Normal	29	32	24	17	3	1	0.005	0.096
	Abnormal	0	1	0	0	0	1		

## Data Availability

The data used to support the findings of this study are included within the article.
